# The impact of varying dextran oxidation levels on the inhibitory activity of a bacteriocin loaded injectable hydrogel

**DOI:** 10.1007/s13346-022-01201-x

**Published:** 2022-07-18

**Authors:** James Flynn, Mario Culebras, Maurice N. Collins, Sarah P. Hudson

**Affiliations:** 1 Department of Chemical Sciences, Bernal Institute, SSPC – The SFI Pharmaceutical Research Centre, University of Limerick, Limerick, Ireland; 2 School of Engineering, Stokes Laboratories, Bernal Institute, University of Limerick, Limerick, Ireland; 3 Health Research Institute and AMBER, University of Limerick, Limerick, Ireland

**Keywords:** Controlled release, Bacteriocins, Drug delivery, Hydrogel, Antimicrobial, Polysaccharides

## Abstract

**Abstract:**

In the design of injectable antimicrobial dextran-alginate hydrogels, the impact of dextran oxidation and its subsequent changes in molecular weight and the incorporation of glycol chitosan on (i) gel mechanical strength and (ii) the inhibitory profile of an encapsulated bacteriocin, nisin A, are explored. As the degree of oxidation increases, the weight average molecular mass of the dextran decreases, resulting in a reduction in elastic modulus of the gels made. Upon encapsulation of the bacteriocin nisin into the gels, varying the dextran mass/oxidation level allowed the antimicrobial activity against *S. aureus* to be controlled. Gels made with a higher molecular weight (less oxidised) dextran show a higher initial degree of inhibition while those made with a lower molecular weight (more oxidised) dextran exhibit a more sustained inhibition. Incorporating glycol chitosan into gels composed of dextran with higher masses significantly increased their storage modulus and the gels’ initial degree of inhibition.

**Graphical abstract:**

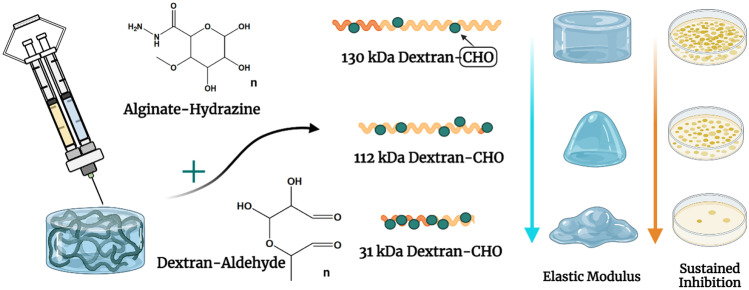

**Supplementary information:**

The online version contains supplementary material available at 10.1007/s13346-022-01201-x.

## Introduction

Infectious diseases were the world’s leading causes of death in the 1900s. Since then, antibiotics have played a major role in improvements of human health, wellbeing and life expectancy globally [1]. Drug-resistant bacteria have been the cause of an increasing number of deaths in recent years, with approximately 25,000 deaths in Europe and 700,000 globally per annum [2, 3]. The rise in antimicrobial resistance cases has been accelerated by multiple factors including, but not limited to, the inappropriate use of antibiotics, and genetic factors intrinsic to bacteria [4], posing major threats to public health, mortality and morbidity [5].

Agencies such as the Infectious Diseases Society of America and the World Health Organisation have highlighted the unique threat of a clique of microorganisms that bypass the biocidal action of antibiotics, representing new paradigms in the spread of resistance cases [6]. These ESKAPE pathogens are a nosocomial collection of *Enterococcus faecium*, *Staphylococcus aureus*, *Klebsiella pneumoniae*, *Acinetobacter baumannii*, *Pseudomonas aeruginosa* and *Enterobacter* spp. [7]. Analysis of the Clinical and Laboratory Standards Institute guidelines by Mulani et al., in 2019, found that many antibiotics suggested against ESKAPE pathogens since 2010 have become ineffectual, with few antibiotics or antibiotic combinations to replace them, added [8, 9]. Novel antimicrobial therapeutics, such as antimicrobial peptides (AMPs), may have the potential to provide a viable alternative to current ineffectual antibiotics, and short cationic peptides offer a potent alternative to longer, natural AMPs [10–12].

AMPs are characterised by their overall positive charge, and hydrophobic amino acid residues [9, 11]. Bacteriocins are typically low molecular weight AMPs, produced by bacterial strains to inhibit competing strains in the same ecosystem competing for nutrients. A number of bacteriocins have been identified with activity against clinically relevant Gram-negative and Gram-positive bacteria, including bacterial strains that show resistance to commonly used antibiotics [13–15]. Thus, bacteriocins are a favourable alternative to traditional antibiotics. A recent review from our group on the issues surrounding the development of bacteriocins into clinical therapeutics highlighted issues such as their poor physicochemical characteristics, and highlighted the potential of polymeric gels in the formation of delivery matrices for bacteriocins to improve their activity and stability [16]. To date, a variety of platforms have been studied for the delivery of these biologics, such as mesoporous materials [17, 18], solid lipid nanoparticles [19, 20], micro and nanoparticles [21–23], and suspensions—for administration as enema preparations [24] and for subcutaneous administrations [25–28].

Hydrogels are water-swollen polymeric networks which maintain a distinctive three-dimensional structure. In fact, they were the first biomaterial to be used in the human body and were traditionally synthesised by copolymerisation, reactive polymer precursors or cross-linking via polymer–polymer reactions [29]. It is the high water content of hydrogels that makes them so favourable for biomedical applications and aids in their biocompatibility in vivo [30]. Their viscoelastic nature reduces damage to surrounding tissue when implanted into the host [31]. Additionally, their mechanical properties can be tailored to parallel those of living tissue [31]. Biodegradable hydrogels are important biomaterials for both tissue engineering and drug delivery applications [32, 33]. Hydrogels derived from natural proteins and polysaccharides make for ideal tissue engineering scaffolds, as they resemble the extracellular matrices of many types of tissue, comprising of various amino acids and sugar-based macromolecules [34]. In situ cross-linking mechanisms can render hydrogels suitable for injection [35].

Hydrogel formation via covalent cross-linking of reactive species offers the ability to develop highly tuneable 3D networks for a variety of applications from cell culturing to drug delivery [32, 36–38]. Oxidation via reaction with sodium periodate (NaIO_4_) is a well-documented mode of chemical functionalisation [39], and controls the degree of oxidation, thus providing a means of changing the materials mechanical and chemical properties. Hudson et al. previously reported that dextran-aldehyde conjugated with amphotericin B and carboxymethyl cellulose functionalised with hydrazine groups covalently cross-linked in situ to form antifungal hydrogels. The degree of aldehyde substitution was varied by changing the mol% of sodium periodate (NaIO_4_) used, where lower cross-link densities showed lower release of amphotericin B with lower degrees of aldehyde functionalisation [40]. The release of GLP-1 and BSA was studied from PEG-DA gels with varying cross-link density degrees in studies by Bal et al. [41]. It was found that lower cross-link densities showed higher levels of release, presumably due to the shorter diffusion paths available within the gel mesh [41]. Demirci et al. showed hydrogels with varying cross-link densities, composed of the polysaccharide levan from *Halomonas* covalently cross-linked with butanediol diglycidyl ether also for the delivery of amphotericin B. The cross-link density was varied by changing the ratio of levan to ether [42]. A paper by Zamboni et al. varied cross-link density by changing the concentration of the cross-linker bis(B-isocyanatoethyl) disulphide, to develop gels tuneable in their physiochemical and immunomodulatory properties [43].

In previous studies from our group, the swelling and strength of dextran-aldehyde and alginate-hydrazine in situ forming injectable hydrogels were controlled through the substitution of the functionalised alginate with an ethylene glycol functionalised chitosan polymer [44]. Here, the dextran molecular weight is varied by controlling the degree of oxidation, whilst retaining a constant cross-link density via an excess of aldehyde groups on the dextran, relative to the number of hydrazine groups on the alginate. The aim of this current study was to determine if changing the degree of dextran oxidation, and thus the molecular weight of the dextran, would allow further control of the properties and behaviour of nisin within the gel network, to achieve a tunable inhibitory profile of the encapsulated nisin. The impact of these changes on the strength, stiffness and degree of swelling of the gel, and on the antimicrobial activity of the gels upon encapsulation of nisin, is investigated. In an attempt to vary the viscoelasticity of the formed gels, the incorporation of the more elastic, higher Mw biopolymer, glycol chitosan, may increase the gel storage modulus, extending the durability of these gels. Thus, it is postulated that variation of the weight average molecular weight of the dextran by varying its degree of oxidation, and incorporation of a water soluble chitosan, may allow for modulation of the gel elastic modulus and infer antimicrobial tunability to the gel.

## Materials and methods

### Materials

Nisin A (95%, isolated from *Lactococcus lactis* in sauerkraut) was obtained from Handary, Belgium. Dextran (from *Leuconostoc mesenteroides*, 100–200 kDa), dextran 60 (from *L. mesenteroides*, ~ 60 kDa), alginic acid (from brown algae, low viscosity (4–12 cps), M/G ratio 1.43 as determined by ^13^C SS-NMR below), sodium (meta) periodate (> 99%), ethylene glycol (99%), N-hydroxysulfosuccinimide sodium salt (S-NHS, > 98%), N-(3-dimethylaminopropyl)-N′-ethylcarbodiimide hydrochloride (EDC, crystalline), adipic acid dihydrazide (ADH, > 98%), hydroxylamine hydrochloride, glycol chitosan (degree of deacetylation: > 60%), trifluroacetic acid (TFA, > 99%), acetonitrile (ACN, > 99.9%), phosphate-buffered saline (tablets, PBS), and sodium dodecyl sulphate were all purchased from Sigma-Aldrich, Ireland. Dimethyl sulfoxide (DMSO, for molecular biology) was purchased from VWR International. Liquid nitrogen was supplied by BOC Gases (Ireland). Glycine (lab grade), 2,4,6-trinitrobenzene sulfonic acid (5% w/v, MeOH), potassium chloride (99%) and hydrochloric acid (37%, fuming) were purchased from Fisher Scientific, Ireland. Pullulan/dextran standards for light scattering/tetra detection (TDS3030) were provided by Particular Sciences, Dublin, Ireland. *Staphylococcus aureus* (DSM 20231) was purchased from the Leibniz Institute DSMZ-German collection of Microorganisms and Cell Cultures.

### Methods

#### Functionalization of polymers

Dextran was oxidized to yield aldehyde groups as reported by Flynn et al. [44]. A 1% w/v dextran solution was prepared in deionised water (diH_2_O). An aqueous suspension of sodium periodate at varying concentrations (0.802 g (0.37 mol%), 1.604 g (0.75 mol%) or 3.208 g (1.5 mol%) — corresponding to low, medium and high oxidation levels respectively) was added to the dextran solution slowly while stirring vigorously. The solution was stirred for 2 h in the dark. After the 2-h period, ethylene glycol was added to stop the reaction (0.27% v/v). The solution was stirred for an additional hour, after which it was dialyzed against a 3.5 kDa molecular weight cutoff (MWCO) cellulose membrane in 5L diH_2_O with 6 water changes over 3 days, followed by flash freezing with liquid nitrogen and bulk freeze drying (−85 °C). The oxidized dextran is referred to as Dex_DO%_ when used in gel formation, where DO refers to the degree of dextran oxidation. Dextran 60 was functionalised in the same way, using the same mol% NaIO_4_ as the medium DO dextran (1.604 g or 0.75 mol%).

A solution of alginic acid was prepared in diH_2_O (1% w/v) by dissolving 500 mg alginic acid in 50 ml diH_2_O and stirred for 1 h to dissolve fully. Meanwhile, 152 mg S-NHS and 134 mg EDC were dissolved separately in 2 ml DMSO (aqueous, 50%). ADH was added (3.96% w/v) to the alginate solution and the pH was adjusted to 6.8 with 1 M NaOH. The EDC and S-NHS solutions were added, and the solution was allowed to stir for 6–8 h, with the pH being constantly adjusted to 6.8 as necessary. The solution was dialyzed against 10 kDa MWCO cellulose membrane in 5L diH_2_O with 6 water changes over 3 days and freeze dried.

#### Characterisation of uncross-linked polymers

##### Polymer oxidation/functionalisation

The degree of dextran oxidation (DO) was determined using a method reported by Zhao and Heindel [45]. A 0.25-M solution of hydroxylamine hydrochloride was prepared in diH_2_O. Dex_DO%_ was dissolved in the solution (0.4% w/v). 0.1 M NaOH was added in 100 µl volumes until the rate of change of pH remained constant (equilibrium). The degree of oxidation was determined using Eq. (1) below, where DO is the degree of oxidation (%), *V* is the volume of NaOH required to reach equilibrium (L), *C* is the concentration of NaOH (M), *n* is the theoretical number of aldehyde groups per repeating unit (2), *m* is the mass of dextran added (0.1 g) and *M* is the molecular weight of a single glucose unit.

The degree of alginate functionalisation with hydrazine groups was determined using the 2,4,6-trinitrobenzene sulfonic acid assay (TNBSA), based on a procedure by Hermanson [46]. A standard curve of glycine was prepared at concentrations ranging from 5 to 20 µg/ml, in a fresh 0.1 M sodium bicarbonate buffer (NaHCO_3_, pH 8.5). Alginic acid and alginate-hydrazine solutions were also prepared in 0.1 M NaHCO_3_ at concentrations of 0.35 mg/ml. A 5% w/v solution of TNBSA (in MeOH) was diluted to 0.01% v/v in 0.1 M NaHCO_3_ buffer (prepared and used fresh). To 500 µl of each sample, 250 µl of 0.01% TNBSA was added and samples were mixed well by vortex. The samples were incubated for 2 h at 37 °C, static. After incubation, 125 µl HCl (1 M) was added to each sample and vortexed. Samples were transferred to quartz cuvettes and analysed spectrophotometrically in a Shimadzu UV Spectrophotometer (UV-1800) at 335 nm. The concentration of hydrazine groups in the alginate-hydrazine was determined based on the stoichiometric ratio of primary amines per glycine molecule using the standard curve of glycine (in 0.1 M NaHCO_3_). Blanks were run in conjunction and subtracted from spectrophotometric readings at 335 nm.

1 $$\mathrm{DO}=\frac{{V}_{\mathrm{NaOH} }\times {C}_{\mathrm{NaOH}}}{{n}_{C=O} \times \frac{m}{M}} \times 100$$

##### Molecular structure characterisation

The molecular weight, number average molecular weight and polydispersity of the different functionalized dextrans (low, medium and high DO) and alginate, and the as-received dextran, dextran 60 and alginate, were determined using gel permeation chromatography on an ÅKTA Pure 25 (Cytiva) chromatography system and Malvern Reveal (Particular Sciences, Ireland) multidetector system. The system was calibrated with pullulan standards (1 mg/ml, 50 mM sodium sulphate (Na_2_SO_4_)) and verified with dextran standards (2 mg/ml, 50 mM Na_2_SO_4_). Dextran samples (2 mg/ml) were run using a 50 mM Na_2_SO_4_ mobile phase, with a flow rate of 0.2 ml/min and sample volume of 100 µl with a dn/dc of 0.147. Alginate samples were run with a 50 mM Na_2_SO_4_ mobile phase, at a flow rate of 0.4 ml/min with a sample volume of 100 µl and a dn/dc of 0.15 [44]. Glycol chitosan samples were prepared and run using 0.1 M Na_2_SO_4_ prepared in 0.5 M acetic acid, with a sample volume of 100 µl and dn/dc of 0.15 [44]. Dn/dc values were obtained from the Malvern Reveal instrument library. All samples were filtered through 0.2 µm PES filters and run through a TSKgel SuperMultipore PW-H (8 µm particle size) column and subsequently through a TSKgel SuperMultipore PW-M (5-µm particle size) column in series (Tosoh Bioscience), with detectors at 30 °C [47]. The mannuronic acid/guluronic acid ratio (M/G ratio) of the as-received alginate was directly quantified from a ^13^C CP-MAS NMR spectrum (supplementary information) of the polymer. This was done by calculating the ratios of ^13^C peak intensities from the mannuronic acid and guluronic acid components as described by Salomonsen et al. [48].

#### Formation of covalently cross-linked gels

Hydrogels were formed using a combination of 60 mg/ml Dex_DO%_ or Dex60_DO%_ dissolved in KCl/HCl (pH 2), and 30 mg/ml alginate-hydrazine dissolved in PBS (pH 7.4) [44]. For gels containing nisin, the functionalised dextrans (60 mg) were dissolved in 1 ml of a 20 mg/ml solution of nisin (in KCl/HCl, pH 2), such that each 100 µl gel would contain 1 mg nisin. Gels with varying dextran DO were injected from a double barrel syringe with alginate hydrazine in one 1-ml syringe and Dex_DO%_/Dex60_DO%_ in the other, through a 21-G needle into a mould (volume, 100 µl), and allowed to set for 20–30 min at 37 °C. Gelation occurred within 2–10 s of injection into the mould. To form gels with glycol chitosan incorporated, a 30 mg/ml alginate-hydrazine solution was prepared in PBS. Glycol chitosan (3% w/v) was added once the alginate-hydrazine was dissolved and stirred at 350 rpm at 37 °C. The alginate-hydrazine/glycol chitosan solution was used as above to prepare gels containing both nisin and GC.

#### Characterisation of hydrogels

Mechanical characterisation of the gels both with and without nisin was carried out using a custom built compression test rig. The cross-sectional area (CSA) of the gels was determined by measuring the diameter using a callipers and calculating CSA using π(D/2)^2^. A pressure of 100 kPa was applied to the gel (relative to the area of each sample), at a speed of 0.25 µm/min. The Young’s modulus (*E*) was determined based on the stress strain curve generated from the compression data. Dynamic mechanical analysis (DMA) was carried out in a TA Instrument Q800 to determine the storage (*G*′) modulus of the different gels. The samples were analysed in a compression geometry using the multi-strain mode with an oscillatory deformation of 10 µm and a frequency of 1 Hz at static forces in the range of 0.001–0.6 N (depending on the hydrogel strength). The swelling and stability of the gels were tested under both low pH conditions in KCl/HCl (pH 2) and phosphate-buffered saline (PBS, pH 7.4). Gels were submerged in 1 ml of buffer, and at specified time points gels were carefully removed, blotted dry with filter paper, and weighed. Media was replenished totally (1 ml) at each time point. The study was carried out at 37 °C, with no shaking/rocking.

#### In vitro antimicrobial activity assay and in vitro release of nisin

An antimicrobial activity study was carried out in conjunction with the release study at pH 2, which represents the optimum solubility of nisin [49] (gels submerged in 1 ml KCl/HCl, pH 2 at 37 °C). At time points of 15, 30, 45, 60 min and up to 3 h on day 1, 1 ml of KCl/HCl (release sample) was totally removed and replenished with a fresh 1-ml aliquot of KCl/HCl. This was continued once daily up to day 8. One hundred microliters of each release sample was taken and added to the wells of 24-well assay plates, without filtering. The plates were UV irradiated for 30 min with the samples added, after which 300 µl of an overnight culture of *S. aureus* in BHI broth, diluted to an OD_595nm_ of 0.1, was added to each sample. Controls of 100 µl KCl/HCl and 300 µl culture were run alongside release samples, as well as release samples from gels containing no nisin. The plates were covered and incubated at 37 °C, shaking at 90 rpm for 4 h. Viable colonies were counted by means of the single drop plate method [50]. Serial dilutions were prepared in PBS (sterile, pH 7.4) after incubation. 10 µl of each sample was applied to BHI agar plates in drops, and dilutions of 10^−3^, 10^−5^, 10^−7^ and 10^−9^ were plated. In certain instances, samples were repeated and plated neat or at a dilution of 10^−1^. Plates were incubated overnight at 37 °C (~ 88% RH). Colonies were counted and expressed in log colony-forming units per millilitre, log CFU/ml (*n* = 3). The results of the antimicrobial activity assay were used to determine the concentration of nisin released from the gels at each time point, based on a standard curve generated from the inhibitory data (supplementary information).

#### Statistical analysis of data

Analysis of variance (ANOVA) was utilised where needed for the determination of variance and thus statistical significance of different samples. ANOVA was carried out using Excel 2016 (Microsoft Office Professional Plus, V16), through the ‘Data Analysis Tool’ in the ‘Analysis’ Excel add in. In all applicable cases, an α of 0.05 (5%) was used for ANOVA. All analyses were carried out in triplicate at minimum. Standard sample deviations were determined using the Microsoft Excel 2016 ‘STDEV.S’ function.

## Results and discussion

### Polymer characterisation

The physicochemical properties of the polymers used in the hydrogels in this study are given in Table 1. Oxidation of dextran with a mol% of NaIO_4_ of 0.37% was determined to be 14% ± 1, implying that 14% of the dextran subunits were oxidised. A mol% NaIO_4_ of 0.75% yielded dextran aldehyde with an oxidation level of 34% ± 8, and a mol% NaIO_4_ of 1.5% had a DO of 79% ± 1, all determined by titration of the functionalised dextran in hydroxylamine hydrochloride (0.25 M) against 0.1 M NaOH (Eq. 1). The degree of oxidation of the lower molecular weight dextran used, Dex60, functionalised with a mol% NaIO_4_ of 0.75%, was determined to be 15% ± 1.

**Table 1:** Data from the characterisation of the polysaccharides and GC, where *x*% subscript indicates the degree of oxidation

**Sample**	**Mw (kDa)**	**DO (%)**	**PDI (Mw/Mn)**	**IV (dL/g)**
Alginate as received [44]	49.1 ± 0.2	–	1.44	2.14 + 0.01
Alginate-hydrazine [44]	39.1 ± 3.7	–	1.03	1.26 + 0.06
Glycol chitosan [44]	125.2 ± 2.2	–	1.06	1.17 + 0.08
Dextran as received	204.6 ± 7.9	0.9	1.52	0.34 + 0.03
Dex_14%_	130 ± 7	14 (low)	1.24	0.28 + 0.07
Dex_34%_	112.2 ± 17	34 (medium)	1.32	0.28 + 0.03
Dex_79%_	30.9 ± 16	79 (high)	1.20	0.25 + 0.04
Dextran60 as received	72.6 ± 0.6	0.8	1.10	0.26 + 0.01
Dex60_15%_	58.3 ± 1.7	15 (low)	1.10	0.22 + 0.01

The molecular weight of alginic acid, functionalised and as received was characterised in a recent study [44]. Briefly, the Mw of the as-received alginic acid was determined to be 49.1 ± 0.24 kDa, with the Mw of the functionalised alginate-hydrazine being 39.1 ± 3.7 kDa. The ratio of mannuronic acid to guluronic acid groups in the as-received alginic acid was found to be 1.43 (supplementary information). Glycol chitosan was also characterised in this previous study, where the Mw was determined to be 125 ± 2.2 kDa, with a medium size distribution [44].

### Characterisation of hydrogels

**Fig. 1:**
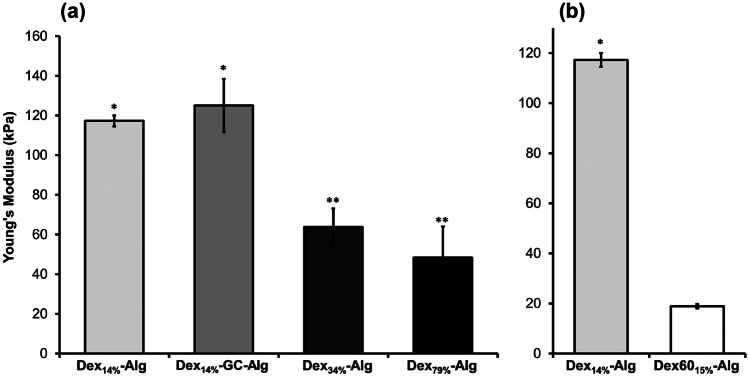
Young’s modulus (*E*) for the (a) Dex14%-Alg, Dex14%-GC-Alg, Dex34%-Alg and Dex79%-Alg gels, and (b) Dex_14%_-Alg and Dex60_15%_-Alg gels. Gels contained 1 mg nisin each, based on three independent samples (*n* = 3). * and ** denote samples where the difference in Young’s modulus is not statistically significant (α > 0.05)

**Fig. 2:**
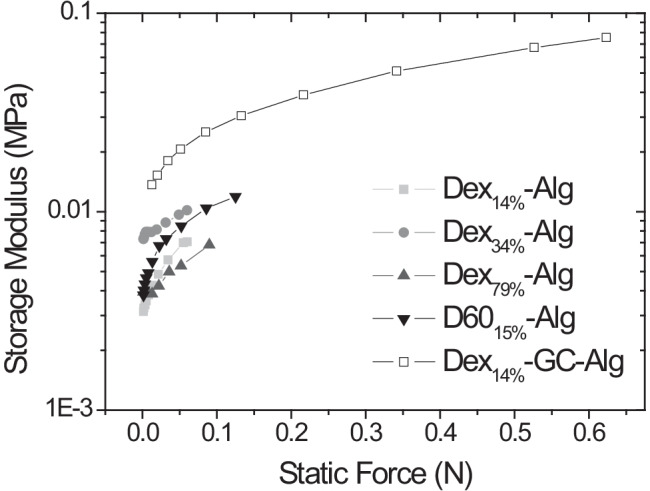
Storage (*G*’) modulus of the Dex_14%_-Alg, Dex_34%_-Alg, Dex_79%_-Alg, Dex_14%_-GC-Alg and Dex60_15%_-Alg gels, as obtained by dynamic mechanical analysis

The gels with the highest initial modulus, ~ 117 ± 0.6 kPa and ~ 125 ± 13 kPa, were the low Dex_DO%_ gels with and without GC, i.e. Dex_14%_-Alg and Dex_14%_-GC-Alg, respectively. Thus, the inclusion of the GC did not cause a significant change in the modulus of the gels (Fig. 1(a)). As the dextran molecular weight decreased, the modulus decreased to 64 ± 9 kPa and 48 ± 15 kPa, for the Dex_34%_-Alg and Dex_79%_-Alg gels, respectively. For gels formed from a lower Mw dextran, Dex60, but with 15% oxidation, a lower modulus of ~ 19 ± 0.8 kPa was found (Fig. 1(b)). The storage modulus increased as a function of the static force for all the different gel formulations which is the trend typically observed in similar hydrogels [51]. The introduction of GC significantly increased the storage modulus (*G*’) of the gels (Fig. 2).

#### Swelling and degradation

**Fig. 3:**
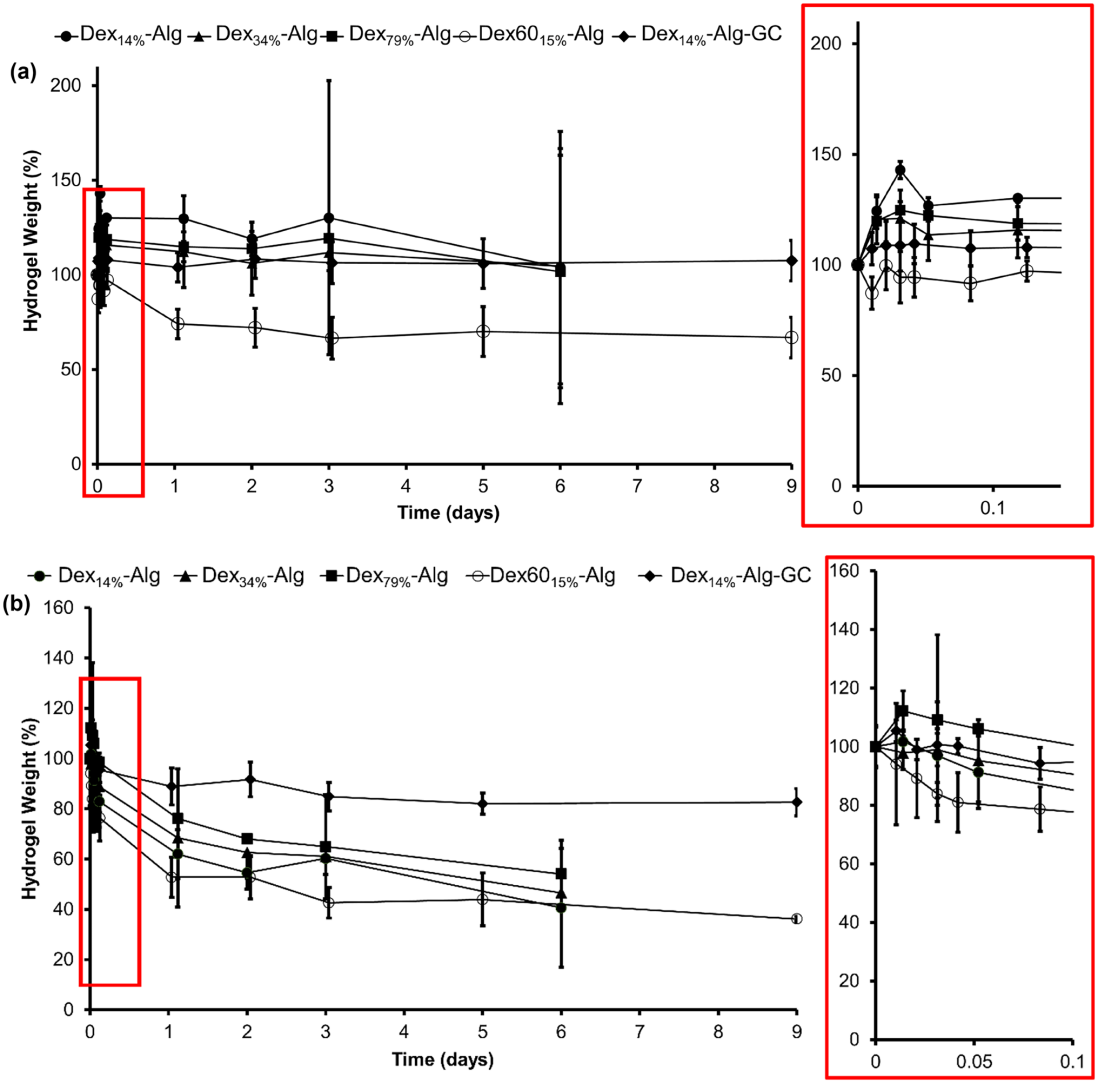
Change in weight of the Dex_14%_-Alg, Dex_34%_-Alg and Dex_79%_-Alg, Dex_14%_-GC-Alg gels, and Dex60_15%_ gels over a period of 6/9 days in **a** HCl/KCl (pH 2, 37 °C) and **b** PBS (pH 7.4, 37 °C). Each data point is based on three independent samples (*n* = 3). No significant difference was observed between the Dex_14%/34%/79%_ gels, at α = 0.05. Final data point indicates disintegration of each respective gel

The degree of swelling and degradation of the hydrogels were characterised at both pH 2 (KCl/HCl) and pH 7.4 (PBS). At pH 2 (37 °C), it was found that the gels gradually degraded over the 6–9-day test, at which point the Dex_14%_-Alg, Dex_34%_-Alg and Dex_79%_-Alg gels disintegrated. No statistically significant difference was observed between these gels (α = 0.05) (Fig. 3a). Incorporation of GC into Dex_14%_-Alg gels appeared to significantly improve stability at pH 2, with 82% of the gel mass remaining after 9 days, in comparison to 40% gel mass after 6 days for Dex_14%_-Alg gels without GC (Fig. 3a). Interestingly, Dex60_15%_-Alg gels also appeared to be more stable at this pH, as evident by the slower time to disintegration, although a similar decrease in mass to the Dex_14%_-Alg, Dex_34%_-Alg and Dex_79%_-Alg gels was evident for the first three days (Fig. 3a). Although not to the same extent, gels in PBS (pH 7.4, 37 °C) also showed a degree of swelling, presumably due to the ionic effect of the salt. As before, however, no statistically significant difference was observed between the Dex_14%_-Alg, Dex_34%_-Alg and Dex_79%_-Alg gels (*p* > 0.05). Dex_14%_-GC-Alg gels swelled to a max of 109% of their original weight after 1 h and showed an increased stability compared to gels without GC, up to 9 days. There was a significant difference in the Dex60_15%_-Alg gels compared to the gels made with the higher mass dextran, whereby they exhibited a higher mass loss in PBS over 9 days (Fig. 3b).

#### Antimicrobial activity assay

**Fig. 4:**
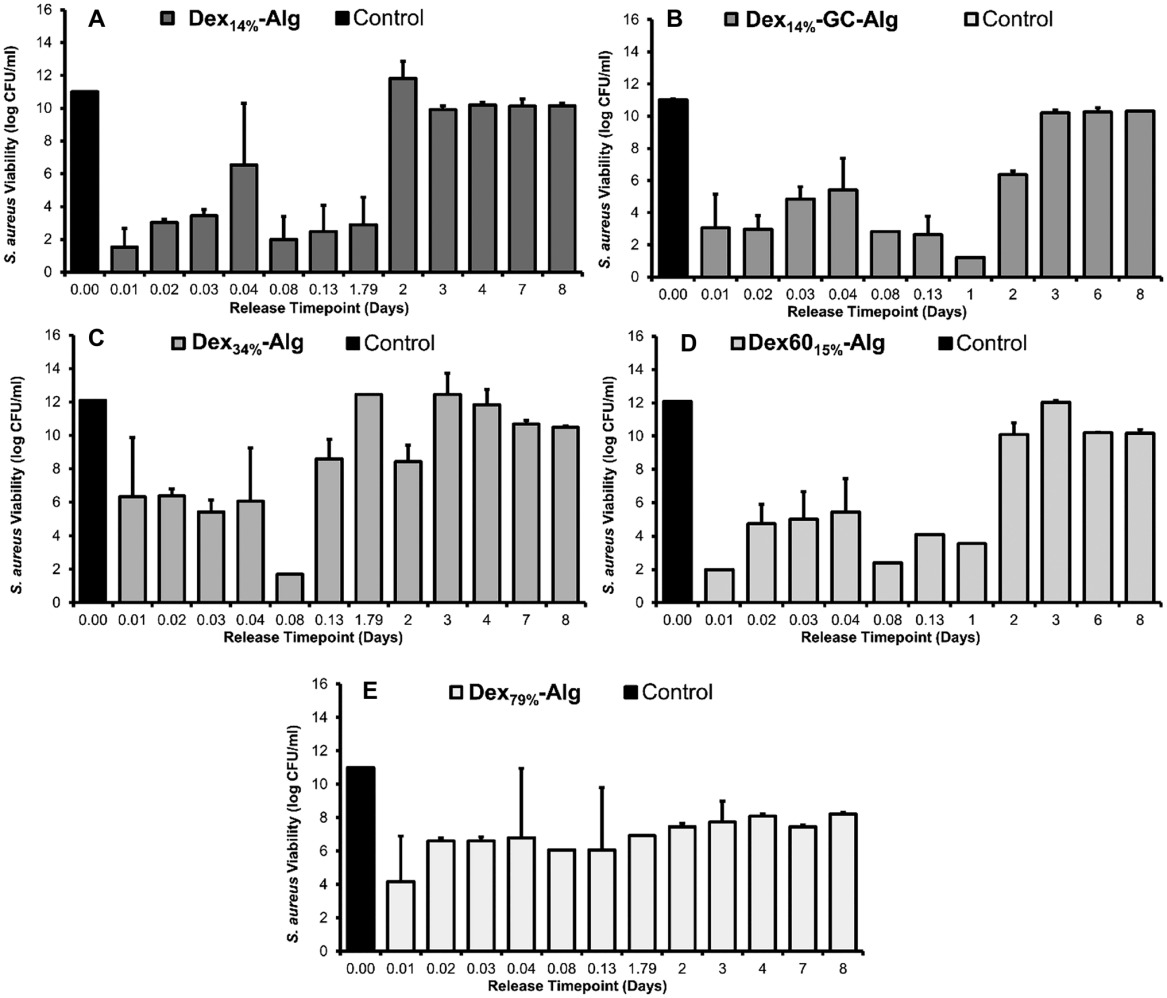
Viability of *S. aureus* (20231 DSM) expressed as log CFU/ml at each time point of the gel release (KCl/HCl, pH 2) up to 8 days, based on three independent samples (*n* = 3), against the buffer control (KCl/HCl) for gels composed of **A** Dex_14%_-Alg, **B** Dex_14%_-GC-Alg, **C** Dex_34%_-Alg, **D** Dex60_15%_-Alg and **E** Dex_79%_-Alg

A concentration/kill curve of nisin A against the growth of *S. aureus* was constructed (supplementary information). Dex_14%_-Alg gels exhibited > 7 log reductions initially with a drop to 4.5 log reductions at 1 h (0.04 days). Another burst of high inhibition was observed (> 7 log reductions) following that up to 2 days, after which the activity subsided (Fig. 4A). As shown above, at 2 days, these gels had lost approximately 50% of their initial mass (Fig. 2). Dex_34%_-Alg gels showed a similar trend with a gradual increase from 5.77 log reductions up to > 7 log reductions in the first 2 h (0.08 days) but inhibition dropped to ~ 3 log reductions by 3 h (0.13 days) with little inhibition observed subsequently, up to 8 days (Fig. 4C). A much more sustained inhibitory profile was observed for the Dex_79%_-Alg gels with an initial > 7 log reduction, followed steadily by 5–6 log reductions up to day 2. This was followed by 2–3 log reductions up to day 8 of testing (Fig. 4E). Gels formed with Dex60_15%_-Alg showed a similar inhibitory profile to the Dex_14%_-Alg gels (Fig. 4D). Formulations mirroring those studied, with nisin excluded, did not show any inhibitory activity over the same testing period (8 days).

#### Calculated in vitro release of nisin from hydrogels

**Fig. 5:**
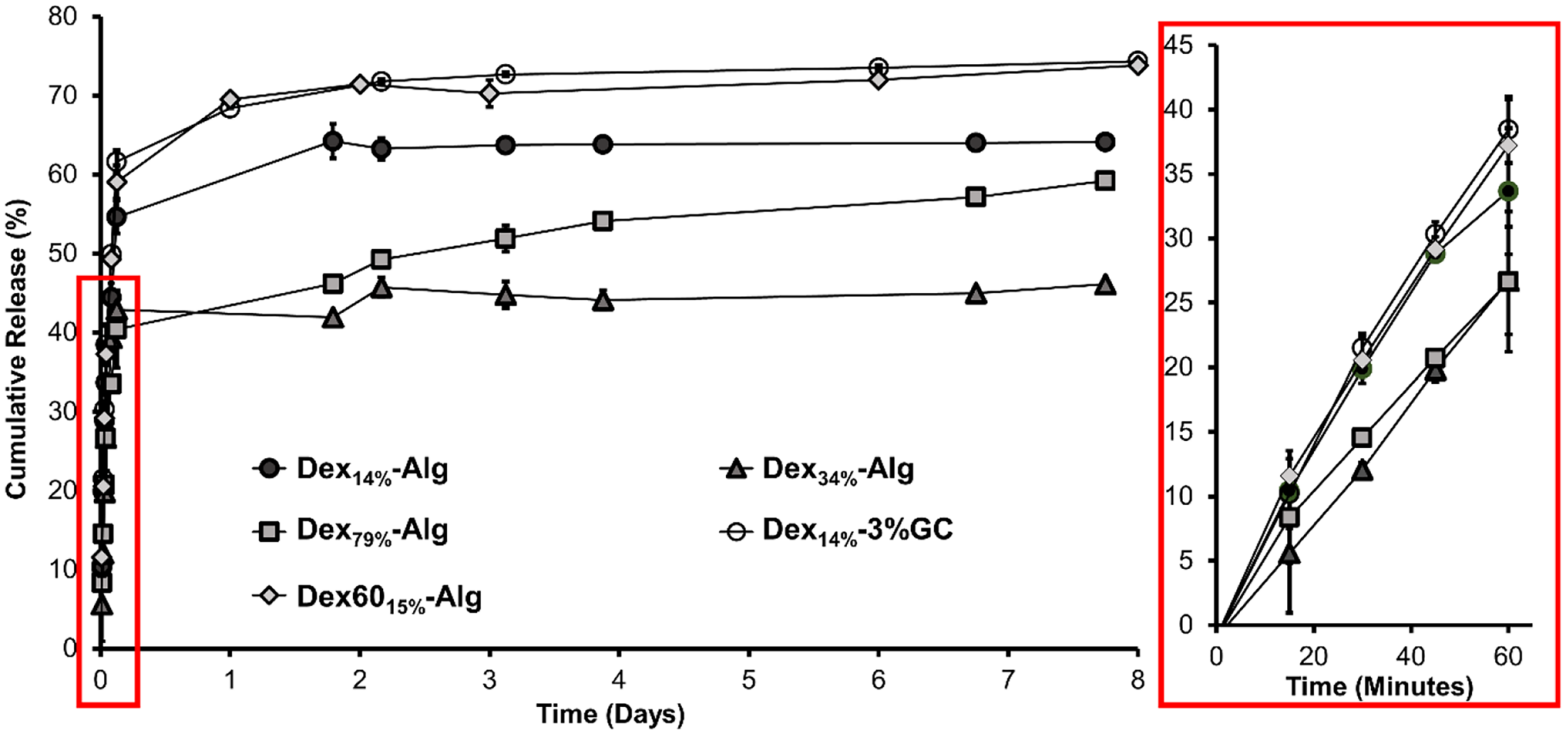
Calculated release of nisin from Dex_14%/34%/79%_-Alg, Dex60_15%_-Alg and Dex_14%_-GC-Alg gels into KCl/HCl (pH 2, 37 °C) calculated based on antimicrobial inhibition, with initial hour release profile inset. Blank gels containing no nisin did not kill and are thus excluded

Overall, the lower DO dextran gels, Dex_14%_-Alg, Dex_14%_-GC-Alg and Dex60_15%_-Alg, showed slightly faster release compared to higher DO dextran gels, Dex_79%_-Alg and Dex_34%_-Alg, over the first 1–2 days, with a maximum calculated release of 74.00 ± 0.02% at day 8. Dex_14%_-Alg gels appeared to release nisin in 10% bursts over the initial 48 h before plateauing at ~ 64% release (Fig. 5). Release was slower from Dex_34%_-Alg gels, with 42% release at 48 h, plateauing at 46.0 ± 0.1% over the 8 days. Dex_79%_-Alg gels showed a similar pattern to Dex_34%_-Alg gels, with release of ~ 5% bursts at each time point reaching 59.0 ± 0.1% at day 8 (Fig. 5). Interestingly, Dex_14%_-GC-Alg gels showed a higher stability in KCl/HCl (Fig. 2) than the gel without glycol chitosan, and also a higher release of nisin, with 10% burst releases in the first 24 h, reaching 74.00 ± 0.02% at day 8 (Fig. 5). The control gel, Dex60_15%_-Alg, showed a maximal release of 74.0 ± 0.1% of nisin while Dex_14%_-Alg gels released only 64.0 ± 0.2% over 8 days. Release data from lower DO gels exhibited non-Fickian diffusion, while higher DO gel release was more indicative of quasi-Fickian release (supplementary information).

## Discussion

As discussed in the ‘Introduction’ section, polysaccharide hydrogels offer a wide range of advantages as drug delivery platforms, from their high biocompatibility, biodegradability, ease of production and the ability to tailor their chemical functional groups. Injectable in situ forming gels allow for potential drug administration locally to a site of infection in vivo. Gels were modified through variation of the hydrogel mesh via the degree of dextran oxidation and its molecular weight. By varying the concentration of periodate used in the dextran oxidation reaction, gels were modified to fine tune their elastic modulus and the resultant release/activity of the bacteriocin, nisin, suspended within the gel. Oxidation of dextran using sodium periodate (NaIO_4_) generates aldehyde (CHO) groups on the dextran repeating units, allowing covalent cross-linking (through hydrazone bonds) with the hydrazine groups on the functionalised alginic acid chains. The cleavage of dextran chains within the oxidation reaction results in a reduced molecular weight. Thus, as the DO increases, the average Mw decreases. It was determined that 1 in every 7.5 repeating units of alginate was functionalised with hydrazine groups, based on the number of primary amines in a glycine standard. Thus, there were approximately five times the number of free aldehyde groups in the oxidised dextran at the lowest degree of oxidation (60 mg/ml, Dex_14%_) compared to the number of free hydrazine groups in the functionalised alginate (30 mg/ml) (supplementary information). All hydrogels formed easily at all levels of dextran oxidation, despite the variation in the molecular weight of the dextran. As the degree of oxidation was increased, the cross-link density was kept constant by the limiting concentration of free hydrazine groups.

Mechanical characterisation showed the structural impact of the varying degree of oxidation (DO), and thus variation of the polysaccharides weight average molecular mass on the strength of the gels as well as their ability to resist elastic deformation. As the change in DO is coupled with changes in the molecular weight, gels formed from dextran polymers with similar levels of oxidation (14–15%) but different molecular masses (130 kDa versus 58 kDa) were compared, Dex_14%_-Alg and Dex60_15%_-Alg respectively. Compression tests were carried out on the five different formulations for comparison purposes to determine Young’s modulus (*E*) of the gels. As the DO was increased, the strength of the gels decreased, presumably due to the change in the degree of polymer chain entanglement due to the decreasing molecular weight of the functionalised dextrans, thus reducing the resistance to elastic deformation (Fig. 1(a)). This was particularly evident in the Dex60_15%_-Alg gels, where a similar DO to the Dex_14%_-Alg gels was utilised, but a significant reduction in modulus was evident for the lower molecular weight dextran (Fig. 1(b)). This control gel demonstrated that the Mw of the dextrans is the dominating factor in Young’s modulus of these gels, rather than electrostatic/Van der Waal forces between the functionalised side groups from the oxidation (C = O). Our group previously showed that addition of glycol chitosan strengthened the gel network and could tune the release of nisin [44]. Here, a hydrogel composed of 3% glycol chitosan and the lowest oxidised dextran (Dex_14%_) was included in the study.

Although the storage and Young’s modulus are related, both magnitudes typically differ due to the way that the load is applied. With the DMA, it is possible to apply a compressive load in a sinusoidal mode allowing the separation of the elastic and viscos component of the material to calculate the storage and loss modulus. Dex_14%_-GC-Alg gels showed the highest values for the storage modulus (*G*’) indicating a stronger elastic component in its polymer network compared to the other formulations. The variation in molecular weight/DO of the dextran component did not have an impact on the storage modulus of the gels, as expected due to the cross-link density being kept constant. The incorporation of GC (3%) affects the viscoelastic properties of the hydrated polymer networks based on Dex-Alg formulations increasing their elastic part due to molecular interactions between the three components (Fig. 2). Substitution of polymers, such as those previously investigated [44], with compounds like glycol chitosan may also provide a means of significantly improving gel viscoelasticity. Swelling and degradation study results indicated that by varying the DO and simultaneously changing the dextran molecular weight, but retaining the cross-link density, the swelling or degradation behaviour of the gels does not change significantly; however, reduction of the mass of dextran used, with the same degree of oxidation, did increase the rate of degradation of the gel.

The in vitro inhibitory activity of all gels was high over the first 2 days (Fig. 4). However, the antimicrobial activity of the Dex_79%_-Alg gels showed a more sustained inhibition over the 8-day testing period than the Dex_14%_-Alg and Dex_34%_-Alg gels (Fig. 4), implying that lower molecular weight coupled with higher DO gives the most sustained killing. The incorporation of 3% GC (w/v) into the Dex_14%_-Alg gels showed a higher degree of log reductions up to day 2, showing a more sustained inhibitory profile than those without GC (Fig. 4B). This could be due to the antimicrobial synergy that nisin and GC exhibit as shown previously, as the release data is based on the inhibitory activity data [44, 49]. HPLC analysis of the released nisin confirmed the retention of the structure of nisin after release from the gels. Thus, it can be deduced that while the average Mw of the polysaccharide has an effect on the strength of the gel (Fig. 1), there was no impact on the antimicrobial activity of the encapsulated bacteriocin (Fig. 4). This suggests that variation of DO offers a sufficient way of achieving prolonged sustained release, without the need to include an additional polymer, likely due to interactions between nisin and the additional carbonyl groups on the polymer chain. Blank gels, containing no nisin, did not inhibit the growth of *S. aureus* over the 8-day testing period. It is important to note that contradictory to Fig. 3, gels did not disintegrate until at least day 8 of testing as the gels were not physically removed and handled throughout the assay.

Nisin release from gels with varying degrees of dextran oxidation was studied in KCl/HCl (pH 2, 37 °C) up to the point of gel disintegration, as nisin exhibits optimum solubility at this pH [49]. Data from the activity assay was used to calculate the nisin release based on the log reductions of *S. aureus* observed (supplementary information). The release could not be directly measured by HPLC due to filtering out of nisin bound to polymer fragments in the release media sample preparation. It was found that varying the DO of dextran controlled the release of nisin from the gels (Fig. 5). It appeared that the molecular weight of the dextran did impact the release of nisin to some extent, with higher release observed when lower mass Dex60_15%_ was used. By varying the dextran DO and molecular weight used in the gels, the target dose of peptide released can be tuned over a desired timeframe. For example, in cases where a high continuous release over a few days may be required, a high molecular weight dextran with a low level of oxidation may suit best. Conversely, for more sustained delivery, low molecular weight dextran with a high level of oxidation would be optimal (Fig. 5). It was hypothesised that the increase in DO of dextran increased the interactions between nisin and the oxidised dextran due to the increased number of aldehyde groups per dextran unit. Higher DO gels showed more diffusion controlled release due to stronger/more interactions of nisin with the aldehyde groups of the oxidised dextran, while the release appeared to be controlled by swelling and degradation in the lower DO gels.

## Conclusion

Varying the degree of oxidation and molecular weight of dextran in an injectable dextran-aldehyde and alginate-hydrazine hydrogel loaded with a bacteriocin allowed tuning of the antimicrobial activity and strength of the gels. Here, a trade-off between increased degree of oxidation (DO) and decreased molecular weight is shown. If the DO of dextran is kept constant and the molecular weight is reduced, the gel elastic modulus is drastically reduced. If the degree of oxidation is increased, and the molecular weight of the dextran simultaneously is reduced, the gel elastic modulus (*E*) also reduces, indicating that molecular weight has a higher influence on gel strength than the degree of oxidation. Incorporation of a stiffer biopolymer, glycol chitosan, provided a means of significantly improving the gel’s viscoelasticity, based on the gel’s storage modulus (*G*’). Increasing the degree of oxidation also leads to an apparent reduction in the rate of nisin released in vitro (KCl/HCl, pH 2), and subsequently showed a sustained antimicrobial activity. Lower degrees of oxidation show a much higher cumulative release of nisin, with high burst release observed in both low and medium levels of dextran oxidation. Thus, variation of the dextran molecular weight and its degree of oxidation in a dextran-alginate covalently cross-linked hydrogel presents a means of varying and controlling the strength and the antimicrobial inhibition profile of a gel containing the encapsulated bacteriocin nisin A. Incorporation of a higher molecular weight biopolymer in tandem allows for enhanced viscoelasticity, opening a broader range of real-world applications for natural hydrogel biomaterials.

## Supplementary information

Below is the link to the electronic supplementary material.

Supplementary file1 (DOCX 276 KB)

## Data Availability

All data is available from the corresponding author upon request.
