# Supplementary material for: The impact of varying dextran oxidation levels on the inhibitory activity of a bacteriocin loaded injectable hydrogel
Source: Drug Deliv Transl Res. 2022 Jul 18;13(1):308–19. doi: 10.1007/s13346-022-01201-x (PMC9726672; doi:10.1007/s13346-022-01201-x)
Supplement: Supplementary file 1 — Supplementary file1 (DOCX 276 KB) [file 13346_2022_1201_MOESM1_ESM.docx]

**The impact of varying dextran oxidation levels on the inhibitory activity of a bacteriocin loaded injectable hydrogel.**

*James Flynn^1^, Mario Culebras^2^, Maurice N. Collins^2.3^ and Sarah P.Hudson*^1^*

*^1^Department of Chemical Sciences, Bernal Institute, SSPC – The SFI Pharmaceutical Research Centre, University of Limerick, Ireland*

*^2^ School of Engineering, Stokes Laboratories, Bernal Institute, University of Limerick, Ireland*

*^3^Health Research Institute and AMBER, University of Limerick, Ireland.*

*Corresponding Author: Sarah.Hudson@ul.ie

**Supplementary Information**

**S.1 Methods**

**S.1.1 ^13^C CPMAS Spectrum of Alginate**

A carbon-13 solid-state nuclear magnetic resonance (SSNMR) spectrum for alginate was acquired on a Bruker Avance III HD NMR spectrometer operating at B0 = 9.4 T, with corresponding ^1^H and ^13^C resonance frequencies of ν0(^1^H) = 400.1 MHz and ν0(^13^C) = 100.6 MHz. Alginate was packed into a 4 mm o.d. zirconia rotor with Kel-F caps under ambient atmosphere, and an experimental ^13^C NMR spectrum was acquired at natural abundance using a 4 mm triple channel (H/X/Y) Bruker MAS probe operating in double resonance mode. The magic angle was optimized using a rotor packed with KBr and spun at 5 kHz. NMR spectra were referenced to TMS at δiso = 0 ppm by setting the high frequency ^13^C resonance in adamantane to 38.48 ppm. The ^13^C CPMAS NMR spectrum was acquired in a single spectral window using the cross-polarization pulse sequence, with a magic-angle spinning (MAS) rotor frequency of 10 kHz, a ^1^H90° pulse width of 2.5 μs, and 50 kHz ^1^H decoupling during acquisition. Proton decoupling was carried out with the SPINAL6466 decoupling sequence at 100%. ^13^C CPMAS spectra were collected using contact time of 2 ms and relaxation delay of 2 s, with 1024 scans collected, as per the recommended method by Salomonsen et al [1].

**S.1.2 Antimicrobial Activity of Nisin and *Staphylococcus aureus***

The inhibitory activity of varying concentrations of nisin against *S. aureus* (DSM 20231) was determined by preparing a stock nisin solution of 1 mg/ml in KCl/HCl (pH 2). A series of nisin concentrations of 10 µg/ml to 30 µg/ml were diluted in PBS and incubated with a culture of *S. aureus* in BHI broth diluted to an optical density (OD_595nm_) of 0.1, at a ratio of 1:3 (1 part PBS with or without nisin, to three parts culture). Controls of *S. aureus* without nisin were run simultaneously. The activity was determined by calculating the log number of colony forming units (per ml) after nisin exposure and subtracting from the log CFU/ml of the control (*S. aureus* and PBS).

**S1.3 Modelling of Calculated Release Data**

The calculated release data was fitted to the Korsmeyer-Peppas (Bruschi, 2015) model to determine the mode of release and the release kinetic constants for each gel, whereby n represents the diffusion exponent and K is the kinetics constant in **Eq. 1** below. Mathematical analysis was carried out using Microsoft Excel (Microsoft Office Professional Plus, V16), sum of squared differences (SSD) were minimised, with constraints on the K and n constants (K and n > 0), using the ‘Solver’ Excel add in. The subsequent correlation of the data and fit was determined using the correlation function in the ‘Analysis’ Excel add in.

$F= \frac{M_{\infty}}{M_{t}}= K_{m}t^{n}$ - **Equation S1** (Bruschi, 2015)

**S.2 Results**

**
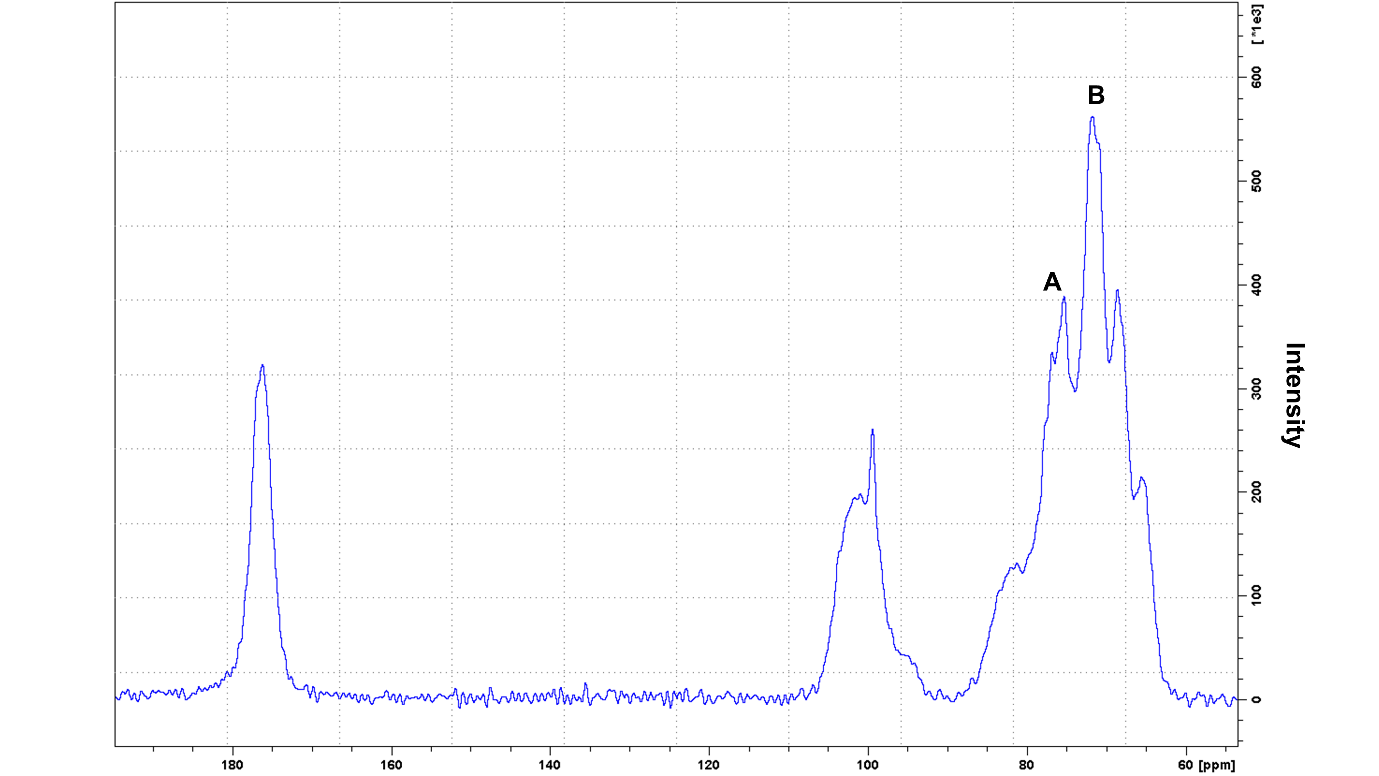
S.2.1 ^13^C CPMAS Spectrum of Alginate**

**Figure S.1** ^13^C SS-NMR spectra of alginic acid where the ratio of the intensity of peak A (5.63 x 10^5^) and B (3.95 x 10^5^) represented the ratio of mannuronic and guluronic acid blocks, as demonstrated in a paper by Salomonsen et al. The ratio was determined to be 1.43.

**S.2.2 Antimicrobial Activity of Nisin A and *Staphylococcus aureus***

**
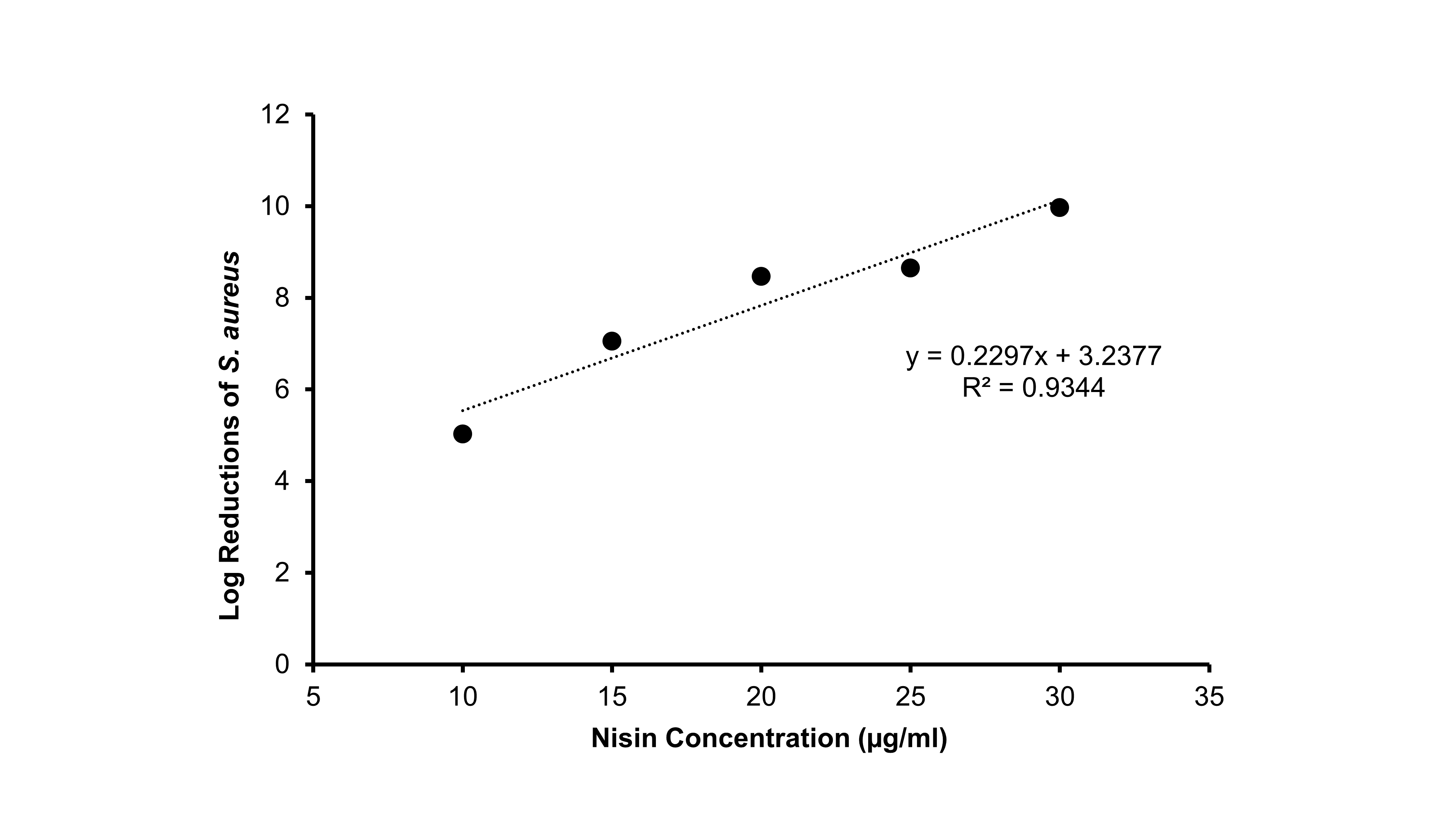
Figure S.2** Graph showing the inhibitory activity of varying nisin concentration on *Staphylococcus aureus* DSM20231 in BHI broth, based on a positive control of *S. aureus* alone (OD_595_ 0.1).

In previous studies it was determined that the median 50% inhibitory concentration (mIC_50_) of nisin against *S. aureus* 20231 DSM was ~ 10-15 µg/ml [2]. Here it was determined that log reductions of 5 in the number of colonies detected were observed at nisin concentrations of 10 µg/ml, increasing up to 8.5 log reductions at 20 µg/ml, and up to 10 log reductions at 30 µg/ml, **Fig. 4**.

**Table S1** Table showing the respective number of repeat units in the gel formulations for Dex-CHO and Alg-ADH. This was used to determine that the hydrazine concentration limited the crosslink density.

| **Polymer** | **Units/Groups** |
| --- | --- |
| Dextran (C_6_H_10_O_5_)n | 3.7 x 10^-4^ repeat units (60 mg/ml) |
| Alginic Acid (C_6_H_8_O_6_)n | 1.7 x 10^-4^ repeat units (30 mg/ml) |
| Dex_14%_ | 5.2 x 10^-5^ units oxidised – thus 1.0 x 10^-4^ CHO groups. |
| Alg-ADH | 1 in 7.5 functionalised – thus 2.1 x 10^-5^ hydrazine groups (limiting reagent) |

**S2.3 Modelling of Hydrogel Release Data**

Gels prepared from lower degrees of dextran oxidation, Dex_14%_-Alg and Dex60_15%_-Alg, including those with GC added, showed diffusivity constants of 0.43 < n < 0.89, indicative of non-Fickian diffusion. Meanwhile higher DO dextran gels Dex_34%_-Alg and Dex_79%_-Alg had lower diffusivity constants (n < 0.45), indicative of quasi Fickian release (Bruschi, 2015), **Table S2**. Mathematical modelling was utilised to further determine the mechanisms of peptide release. This kind of modelling is important considering the array of phenomena involved in drug release such as diffusion and swelling. Models such as zero and first order release models correspond to constant release or release dependent on drug concentration, respectively (Vigata, Meinert, Hutmacher, & Bock, 2020). The Higuchi model initially was designed to model release from ointments, films and planar systems, but now also incorporates 3D dosage forms only where release is diffusion driven (Vigata et al., 2020). The most frequently studied release model for drug release from hydrogels is the Korsmeyer Peppas model (Vigata et al., 2020), **Eq. S1**. In cylindrical shaped gels, release mechanisms can be described as being quasi-Fickian diffusion, where the diffusivity constant n < 0.45 or Fickian diffusion where n = 0.45 while diffusivity constant values of 0.45 < n < 0.89 indicate anomalous or non-Fickian transport (Vigata et al., 2020), indicating that release is principally controlled by degradation and swelling of the gel matrix (non Fickian) (Bruschi, 2015). The higher diffusivity constant 0.62 (**Table S2**), in gels with GC indicate that release is more likely due to swelling (Vigata et al., 2020), which would correlate with swelling/stability studies (**main manuscript, Fig. 3)**.

**Table S2** Table of diffusivity constants and release kinetics, as determined by fitting of the calculated release data to the Korsmeyer Peppas model.

| **Sample** | **D.O** | **K** | **n** | **Type of Transport** | **R^2^** |
| --- | --- | --- | --- | --- | --- |
| **Dex_14%_-Alg** | 14% | 173 | 0.54 | Non-Fickian | 0.99 |
| **Dex_14%_-GC-Alg** | 14% | 241 | 0.62 | Non-Fickian | 0.99 |
| **Dex60_15%_-Alg** | 15% | 194 | 0.56 | Non-Fickian | 0.99 |
| **Dex_34%_-Alg** | 34% | 38 | 0.14 | Quasi Fickian | 0.89 |
| **Dex_79%_-Alg** | 79% | 42 | 0.18 | Quasi Fickian | 0.96 |

**References**

[1] T. Salomonsen, H. M. Jensen, F. H. Larsen, S. Steuernagel, and S. B. Engelsen, "Direct quantification of M/G ratio from (13)C CP-MAS NMR spectra of alginate powders by multivariate curve resolution," (in eng), *Carbohydr Res,* vol. 344, no. 15, pp. 2014-22, Oct 12 2009, doi: 10.1016/j.carres.2009.06.025.

[2] J. Flynn, S. Mallen, E. Durack, P. M. O'Connor, and S. P. Hudson, "Mesoporous matrices for the delivery of the broad spectrum bacteriocin, nisin A," *Journal of Colloid and Interface Science,* vol. 537, pp. 396-406, 2019/03/01/ 2019, doi: <https://doi.org/10.1016/j.jcis.2018.11.037>.
